# High throughput genomic sequencing of bioaerosols in broiler chicken production facilities

**DOI:** 10.1111/1751-7915.12380

**Published:** 2016-07-28

**Authors:** Kate M. O'Brien, Michael S. Chimenti, Morgan Farnell, Tom Tabler, Thomas Bair, Joey L. Bray, Matthew W. Nonnenmann

**Affiliations:** ^1^ Department of Occupational and Environmental HealthUniversity of IowaIowa CityIAUSA; ^2^ Iowa Institute of Human GeneticsBioinformatics DivisionUniversity of IowaIowa CityIAUSA; ^3^ Mississippi State University Extension ServiceMississippi StateMSUSA; ^4^ Department of AgricultureStephen F. Austin State UniversityNacogdochesTXUSA

## Abstract

Chronic inhalation exposure to agricultural dust promotes the development of chronic respiratory diseases among poultry workers. Poultry dust is composed of dander, chicken feed, litter bedding and microbes. However, the microbial composition and abundance has not been fully elucidated. Genomic DNA was extracted from settled dust and personal inhalable dust collected while performing litter sampling or mortality collection tasks. DNA libraries were sequenced using a paired‐end sequencing‐by‐synthesis approach on an Illumina HiSeq 2500. Sequencing data showed that poultry dust is predominantly composed of bacteria (64–67%) with a small quantity of avian, human and feed DNA (< 2% of total reads). *Staphylococcus* sp. *AL1*,* Salinicoccus carnicancri* and *Lactobacillus crispatus* were the most abundant bacterial species in personal exposure samples of inhalable dust. Settled dust had a moderate relative abundance of these species as well as *Staphylococcus lentus* and *Lactobacillus salivarius*. There was a statistical difference between the microbial composition of aerosolized and settled dust. Unlike settled dust composition, aerosolized dust composition had little variance between samples. These data provide an extensive analysis of the microbial composition and relative abundance in personal inhalable poultry dust and settled poultry dust.

## Introduction

Agricultural dust is generated in animal production facilities (Cambra‐López *et al*., 2010). Inhalation exposure to agricultural dust induces pulmonary inflammation and can lead to the development of chronic respiratory diseases (Palmberg *et al*., 1998; Redente and Massengale, 2006; Poole and Romberger, 2012). Several studies have demonstrated that agricultural workers, particularly poultry workers, have a reduction in lung function and higher prevalence of chronic respiratory diseases (*e.g.,* chronic bronchitis) (Zuskin *et al*., 1995; Simpson *et al*., 1998; Radon *et al*., 2001; Rylander and Carvalheiro, 2006; Viegas *et al*., 2013). Furthermore, poultry dust induces pulmonary lesions and cardiac morphological changes in broiler chickens (Oyetunde *et al*., 1978; Riddell *et al*., 1998; Lai *et al*., 2009). Due to these human and animal health implications, it is critical to characterize the composition of poultry dust to determine the source and potential targets for engineering interventions.

Dust generated in poultry production consists of a complex mixture of chicken and human derived dander, bedding, chicken feed, and viable and nonviable microbial populations (Lenhart and Olenchock, 1984; Radon *et al*., 2002). Previous studies have used more specific molecular biology tools such as enzyme‐linked immunosorbent assay and polymerase chain reaction to characterize poultry dust (Kwon *et al*., 2000; Oppliger *et al*., 2008; Prester and Macan, 2010; Just *et al*., 2011; Gerald *et al*., 2014). These studies have shown that poultry dust contains inflammatory agents such as lipopolysaccharide (LPS) and peptidoglycan (PGN), constituents of bacterial cell walls in Gram‐negative and Gram‐positive bacteria (Thedell *et al*., 1980; Sonesson *et al*., 1988; Gerald *et al*., 2014). Also, zoonotic viruses (*e.g*., avian influenza) have been detected in dust (Chen *et al*., 2010; Spekreijse *et al*., 2013). Settled agricultural dust extracts are predominantly employed to determine the mechanism underlying pulmonary toxicity (Palmberg *et al*., 1998; Redente and Massengale, 2006). Furthermore, it is unknown if there is a microbial difference between settled dust and aerosolized dust in large animal facilities.

The advancement of genomic sequencing technology has made the comprehensive analysis of microbes in poultry dust readily available. Previously, pyrosequencing was used to characterize bacteria and fungi targeting ribosomal RNAs (*i.e.,* 16S and 18S), which are ubiquitously expressed in the bacteria and eukaryota domains (Nonnenmann *et al*., 2010; Boissy *et al*., 2014). In one sample of poultry dust, *Staphylococcus cohnii* and *Sagenomella sclerotialis*, respectively, were the most abundant bacterial and fungal species (Nonnenmann *et al*., 2010). Whole‐genome shotgun pyrosequencing demonstrated that there are differing taxonomic profiles and genus abundance among swine, grain, and house dust (Boissy *et al*., 2014). Pyrosequencing technology generally has a high error rate, high cost per megabase, and lack of sufficient sequence coverage needed to assess complex whole genomic samples containing hundreds of low‐abundance bacterial, viral and fungal species. Newer sequencing technology such as the Illumina HiSeq 2500 platform has several advantages over other sequencing technologies. Specifically, the Illumina HiSeq 2500 platform has lower error rates, better breadth and coverage depth, and lower cost per megabase than older technologies. Illumina HiSeq 2500 uses the sequencing by synthesis approach. Briefly, genomic DNA is fragmented into 300–600 base pair segments, and fragments are indexed using adapter sequences. The prepared library DNA is added to a flowcell and undergoes bridge amplification to form clonal clusters, where each cluster represents a single DNA fragment. Four different fluorescent dye‐tagged nucleotides (dNTPs) are added to the reaction. Once the complementary dNTPs are incorporated into the DNA strand, the instrument detects the emission of light at each cluster simultaneously, which allows parallel sequencing of the library. Using paired‐end sequencing, two reads are generated from the same DNA strand by sequencing from both the 5′ and 3′ end.

The aim of this study was to characterize the composition of microbial communities in airborne and settled poultry dust using a whole‐genome shotgun sequencing approach and state‐of‐the‐art analysis tools.

## Results

### Comprehensive analysis of microbial composition of airborne and settled poultry dust in broiler chicken houses

**Figure 1:**
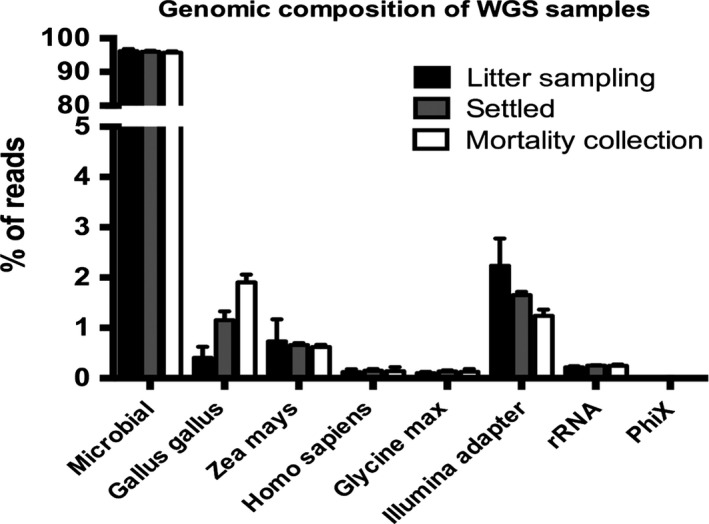
The primary sources of genomic DNA in poultry dust. FastQ Screen was used to determine the percentage of total sequencing reads from microbes, chickens, humans, feed particulates (*Zea mays* and *Glycine max*) and ligated adapters in genomic DNA extracted from poultry dust collected during litter sampling (black), settled (grey) and mortality collection (white).

Microbes account for the vast majority of the extracted DNA from both inhalable and settled poultry dust, yielding 95% of total reads (Fig. 1). DNA belonging to *Homo sapiens* (human) and *Gallus gallus* (broiler chicken) contributed less than 2% of reads in all poultry dust samples. *Zea mays* (maize) and *Glycine max* (soybean) DNA, components of chicken feed, were less than 1% of reads from settled and inhalable dust. Therefore, the vast majority of the DNA reads from each sample represent genuine microbial diversity present in the dust.

**Figure 2:**
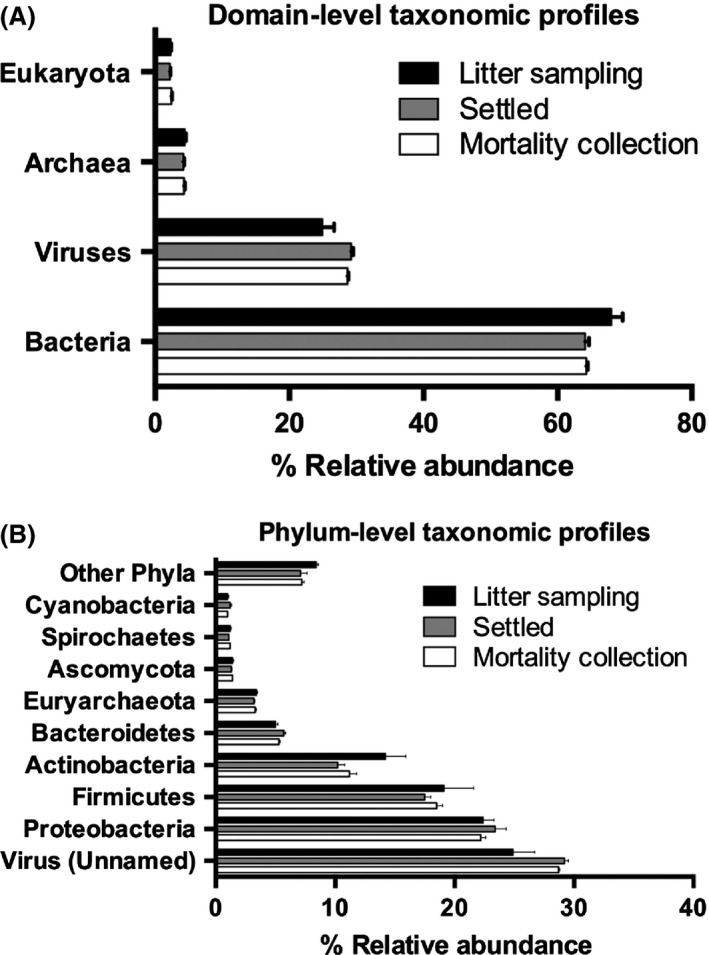
Microbial taxonomic profile of aerosolized or settled poultry dust. Relative abundance of (A) domain and (B) phylum taxonomic profiles in inhalable and settled poultry dust was determined by analysis with the MetaPhlAn2 package. Personal inhalable dust samples were collected during litter sampling (black) or mortality collection (white). Settled dust (grey) samples were obtained from the side‐walls and curtains of the poultry house.

Metagenomic Phylogenetic Analysis 2 (MetaPhlan2) was used as the relative abundance analysis because it reports relative abundance on a community basis (*i.e.,* the proportion of the total number of microbes in the sample belonging to a given species, genus, etc.) rather than on a read basis (Segata *et al*., 2012). This is advantageous because it yields percentages that have greater biological meaning and interpretability than simply the proportion of reads sequenced. MetaPhlan2 analysis showed that inhalable litter sampling (LS) dust, settled (S) dust and inhalable mortality collection (MC) dust had similar relative abundance at the domain level with the majority classified as bacteria (67%, 64% and 64% respectively). Viruses were the second most abundant domain in LS dust (25%), settled dust (29%) and MC dust (29%) (Fig. 2A). Although most viruses in the samples are attributed as ‘no‐name’ or unclassified, there were significant contributions from the plant viral family *Potyviridae* and well‐characterized viral orders *Caudovirales*,* Picornavirales*,* Tymovirales*,* Mononegavirales* and *Herpesvirales* (data not shown).

At the bacterial phylum level, *Proteobacteria* (33%, 37%, 35%), *Firmicutes* (28%, 27%, 28%) and *Actinobacteria* (19%, 15%, 16%) were the most abundant in LS dust, settled dust and MC dust respectively (Fig. 2B). The fungal phylum *Ascomycota* (1.4%, 1.3%, 1.4%) was a smaller relative abundance in poultry dust than bacterial phyla (Fig. 2B).

**Figure 3:**
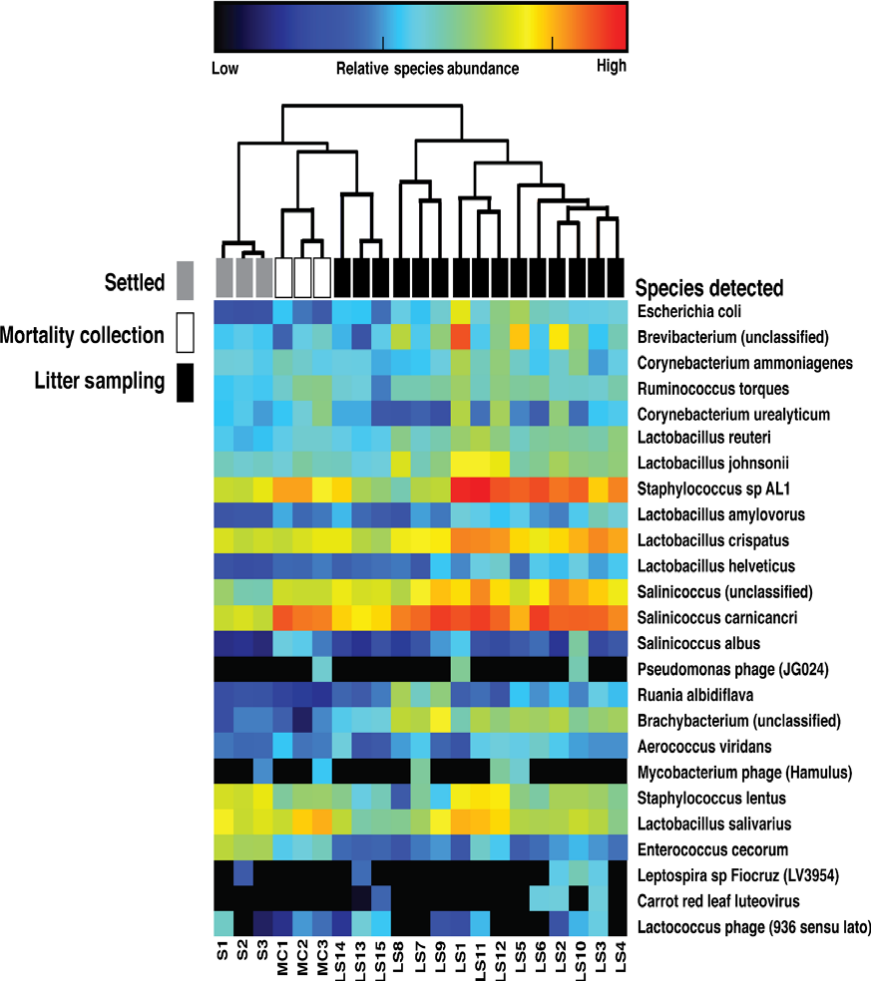
Microbial relative abundance in aerosolized or settled poultry dust. Heat map was generated using MetaPhlAn2 and illustrates the 25 most abundant species in inhalable litter sampling dust (LS1‐15) (white), settled dust (SD1‐3) (grey), inhalable mortality collection dust (MC1‐3) (white) collected on broiler chicken farms. Columns represent the relationship between dust samples, and rows represent the relative abundance of species in the poultry dust.

The heat map in Fig. 3 shows the 25 most abundant species in common among the poultry dust samples. The samples are clustered hierarchically using the Bray‐Curtis dissimilarity method. With the exception of three locations (LS13‐15), the LS dust samples were one distinct cluster. The MC and settled dust samples formed a secondary cluster. *Staphlyococcus sp AL1*,* Salinicoccus carnicancri* and *Lactobacillus crispatus* had a high relative abundance in personal inhalable LS dust (LS1‐15). In comparison, MC dust (MC1‐3) had a reduction in *L. crispatus* but similar relative abundance of *Staphylococcus sp AL1* and *S. carnicancri*. Bacterial genera detected in settled dust (S1‐3) had a moderate relative abundance of *Staphylococcus sp AL1*,* L. crispatus*,* S. carnicancri*,* Staphylococcus lentus* and *Lactobacillus salivarius*. Settled dust had an elevated abundance of *Enterococcus cecorum* compared with LS and MC samples (Fig. 3).

In addition, the viral species, *Lactococcus phage 936* sensu lato, *Carrot red leaf luteovirus*,* Mycobacterium phage Hamulus*,* Pseudomonas phage JG024*, were present at low relative abundance among the poultry dust samples (Fig. 3).

### Bacterial species composition is significantly different between aerosolized and settled dust

**Figure 4:**
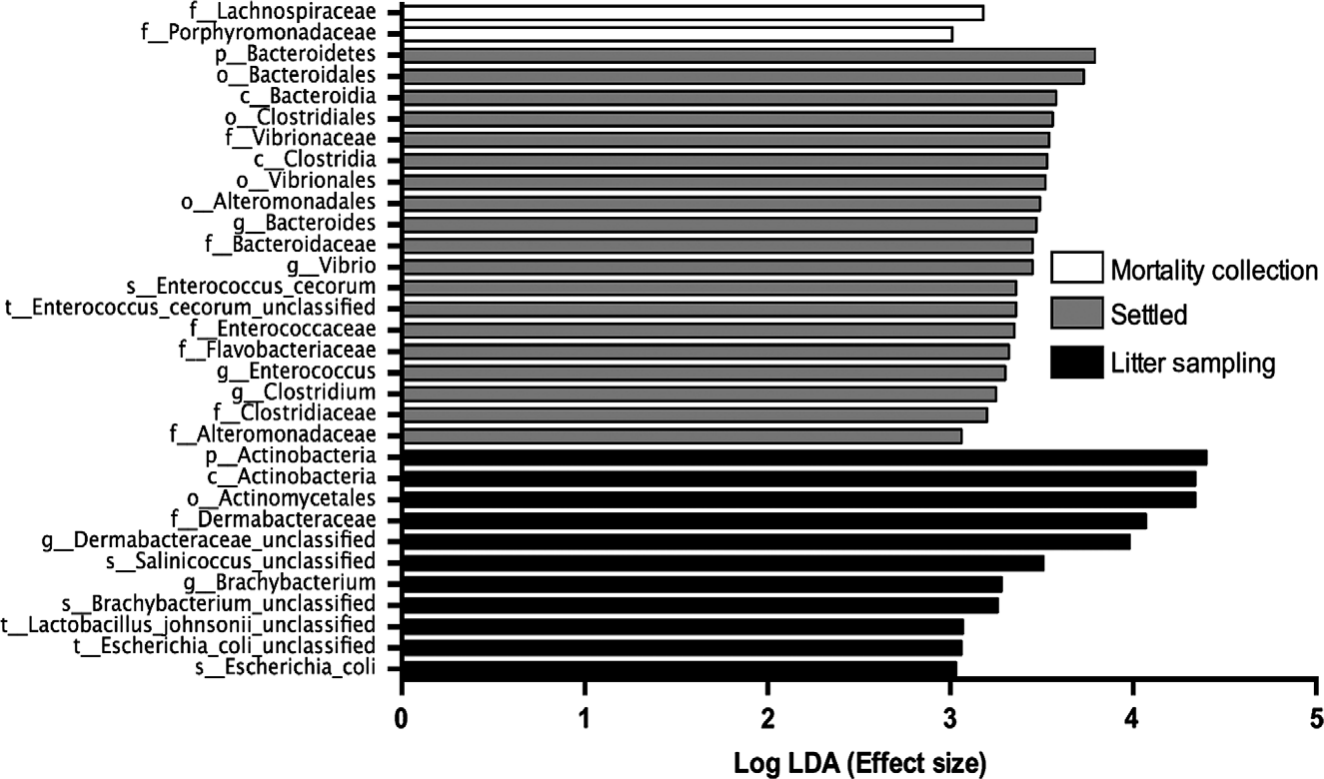
Indicators of either aerosolized or settled dust based on bacterial taxonomical profile. Linear discriminant analysis Effect Size (LEfSe) greater than 3 (*P* < 0.05) was used to illustrate the statistical differences in relative abundances between inhalable litter sampling dust (black), settled dust (grey) and inhalable mortality collection dust (white). The analysis can discriminate based on ‘features’ of the abundance profile including phyla (p), class (c), order (o), family (f), genus (g), species (s) and strain (t).

A statistical analysis of sample relative abundance was performed using the Linear discriminant analysis Effect Size (LEfSe) demonstrated ‘features’ (*i.e.,* taxonomic relative abundances) of samples that may be used as a predictor of either LS, MC or settled dust (Fig. 4). The features shown in Fig. 4 are statistically different between dust types using a Kruskal–Wallis test (*P* < 0.05) and are subsequently found to have the largest effect score in a linear discriminant analysis model between the dust types (log LDA score > 3). Specifically, settled dust samples are characterized by an enrichment in the *Enterococcus*,* Bacteroides*,* Vibrio* and *Clostridium* genera. In contrast, LS dust is characterized by enrichment in the phylum *Actinobacteria,* the genus *Brachybacterium* and family *Dermabacteraceae*. Furthermore, MC dust is enriched in the *Lachnospiricae* family and the *Porphyromonadaceae* family. These taxonomic features could potentially provide reliable ‘biomarkers’ of LS, MC or settled dust in poultry houses. However, further studies are warranted.

### Clustering samples on Bray‐Curtis dissimilarity

**Figure 5:**
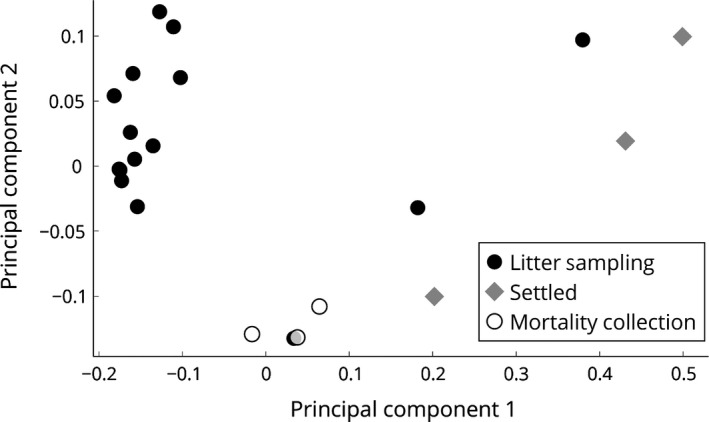
Relationship between the sources of poultry dust. Ordination graph was derived from the Bray‐Curtis dissimilarity matrix calculated in Python. Principal component analysis shows the distances between microbial compositions in inhalable litter sampling dust (black), settled dust (grey) and inhalable mortality collection dust (white). The distance between samples is proportional to their similarity of microbial composition.

The Bray‐Curtis dissimilarity matrix, a statistical test used to quantify the compositional dissimilarity between different ecological sites, was calculated for all samples. Then, a principal component analysis (PCA) was performed to reduce the higher dimensional matrix to allow visualization of the amount of variation between sample profiles (Fig. 5). The LS and MC dust samples showed little variance among the groups, demonstrated by the formation of two distinct clusters. However, LS13‐15 samples are outliers (which could be caused by either batch effects or genuine biological diversity). In contrast, settled dust samples show a greater variance among the three samples.

## Discussion

In this study, next generation sequencing was used to make a comprehensive DNA analysis of poultry dust. Metagenomics analysis of inhalable poultry dust demonstrated that the Gram‐positive bacteria species, *Staphylococcus sp AL1*,* L. crispatus* and *S. carnicancri*, are highly abundant in bioaerosols during LS and MC tasks (Fig. 3). *Staphylococcus sp AL1, L. crispatus* and *Salinicoccus carncancri* belong to the *Firmicutes* phylum, the second most abundant bacterial phylum in poultry dust (Fig. 2B). *Staphylococcus sp AL1* was first isolated from Chinese soy sauce brine fermentation and encodes an *N*‐acyl homoserine lactone (AHL) lactonase gene (Chong *et al*., 2012). AHL lactonases are quorum‐quenching enzymes that inhibits cell‐to‐cell communication of bacteria through the quorum sensing signalling of AHL (Dong and Zhang, 2005). *L. crispatus* has been found to colonize the epithelium of the chicken crop, mature enterocytes at the villus tip of the small intestine, and in the follicle‐associated epithelium (FAE) and Peyer's patches of the ileum (Edelman *et al*., 2002). In addition, *L. crispatus* strain ST1 and 134mi inhibited *E. coli* 789 adherence to the epithelium of the crop and ileal FAE (Edelman *et al*., 2003). *S. carnicancri* is a halophilic bacteria isolated from Korean ‘ganjang‐gejang’, raw crabs preserved in soy sauce, that prefers a salt rich environment of 5–20% (wt/vol) NaCl (Jung *et al*., 2010; Hyun *et al*., 2013). Lu *et al*. (2003) demonstrated that *Salinococcus* spp was present in broiler chicken litter collected from farms in Northeast Georgia using 16S rDNA sequencing. Furthermore, *S. lentus*,* Salinicoccus* spp., *Lactobacillus* spp., *Corynebacterium ammoniagenes*,* Corynebacterium urealyticum*,* Brevibacterium* sp. and *Brachybacterium* spp. were detected in broiler chicken dust and broiler chicken litter (Fig. 3) (Lu *et al*., 2003; Nonnenmann *et al*., 2010). *Staphylococcus* and *E. coli* were the only bacteria detected across studies using culture based and molecular techniques that corresponded to our findings (Brooks *et al*., 2010; Gerald *et al*., 2014). Clearly, the phenomenon of culture bias is limiting the reported compositional diversity of poultry dust in past studies. The results from our study suggest that poultry production facilities have a diverse and highly complex microbial ecosystem which varies substantially between airborne and settled dust.

Both workers and broiler chickens suffer from exposure to dust generated during broiler chicken production. Poultry workers have a higher prevalence of acute and chronic respiratory diseases (*e.g.,* occupational asthma and chronic bronchitis) than any other agricultural occupation, which has been attributed to the induction of the inflammatory response by endotoxin, a constituent of Gram‐negative bacterial cell wall, in organic dust (Clark *et al*., 1983; Simpson *et al*., 1998; Donham *et al*., 2000; Malireddy *et al*., 2013; Viegas *et al*., 2013). For instance, inhalation of LPS, a marker of endotoxin, induced polymorphonuclear neutrophil and myeloperoxidase in the blood and sputum of healthy subjects 24 h post‐exposure (Thorn and Rylander, 1998). In addition, endotoxin was shown to be a predictor of chronic phlegm in poultry workers (Kirychuk *et al*., 2006). For these reasons, an occupational exposure limit of 614 endotoxin units (EU) m^−3^ has been recommended for poultry workers by (Donham *et al*. 2000). However, the inhibition of endotoxin activity in agricultural dust did not attenuate the inflammatory response in lung epithelial cells nor human peripheral blood monocytes (Romberger *et al*., 2002; Poole *et al*., 2010; Gottipati *et al*., 2015). Muramic acid, a marker of the bacterial cell wall component PGN and Gram‐positive bacteria, has a higher concentration in large animal agricultural dust (*i.e., swine and dairy*) than human house dust (Poole *et al*., 2010). Our results further suggest that Gram‐positive bacteria should be used for future mechanistic studies. Furthermore, the vast majority of studies elucidating the agricultural dust induced inflammatory mechanism in the respiratory tract have focused on aqueous or organic settled dust extracts (Poole *et al*., 2010; Gottipati *et al*., 2015). Our results demonstrate a significant difference of microbial diversity and abundance between airborne and settled poultry dust (Figs 3, 4, 5). These results suggest that future *in vitro* and *in vivo* toxicological studies should be performed to differentiate the immunomodulatory effects between bacteria in either airborne dust or settled dust. In addition, this study provides direction for selecting bacteria species as potential targets in future exposure characterization and respiratory toxicological studies.

There are potential limitations that may affect a study of this magnitude and scope. First, batch effects can occur when undertaking a study involving multiple sites spread throughout the Southeast region of the United States. Different environmental and housing conditions may impact the microbial populations present in broiler production in ways that are presently not well understood. For example, variation between samples may be partially due to differences in litter treatment and/or bedding, age of birds, and environmental conditions (*e.g.,* temperature, relative humidity and poultry facility). Although whole‐genome shotgun sequencing is an extremely powerful technique to characterize the ecological diversity of any environment, it cannot distinguish between viable and non‐viable organisms in the sample. Therefore, the relative abundance of viable microorganisms in poultry dust needs to be further evaluated by specific cell culture assays.

Despite these potential sources of variability, we note that there is a robust consistency in the taxonomic profiles reported by the analysis that provides strong support for the general taxonomic profile of poultry production environments as reported. More studies will be needed to assess the role of environmental sources of variability on the taxonomic composition in poultry production environments.

In conclusion, our findings demonstrate that poultry dust is predominately composed of bacteriophage viruses and Gram‐positive bacteria with *Staphylococcus* sp. *AL1*,* L. crispatus* and *S. carnicancri* having the highest relative abundance among the airborne samples. In addition, these results show that the microbial composition of poultry dust varies between airborne and settled dust as well as working conditions. Our results provide an in‐depth analysis of different types of poultry dust which can be used to elucidate mediators of the inflammatory response in poultry workers’ respiratory tract and provide guidance for the development of engineering controls designed for microorganisms present in broiler chicken production facilities.

## Experimental procedures

### Sample population

In this study, we collected either inhalable dust or settled dust samples on commercial broiler chicken farms in the southern region of the United States. Specifically, personal exposure of inhalable dust samples was collected among research staff performing LS (LS1‐15) or MC (MC1‐3) at 16 commercial broiler chicken farms (15 farms:LS and 1:MC). Litter sampling inhalable dust samples were collected among poultry farms during April and May. Seventy‐three percent (11/15) of LS inhalable dust samples and 100% of MC inhalable dust samples had litter bedding treated with sodium bisulphate (PLT) (Table S1). Settled dust (S1‐3) was collected into a 50 ml conical tube (Fischer Scientific, Pittsburgh, PA, USA) from curtains and side‐walls of the chicken house on a university commercial broiler chicken farm during the month of March. This farm had litter bedding treated with liquid aluminium sulphate (Al^+^ Clear A7) (Table S1).

### Sample collection

Two inhalable dust samplers were used for this study, Institute of Occupational Medicine (IOM; SKC, Eighty Four, PA, USA) or Button sampler (SKC). Each sampler contained a polyvinyl chloride filter with diameter of 25 mm and pore size 5.0 μm (SKC). The samplers were located in the personal breathing zone of a research personnel for times raging between 30 and 90 min depending upon the time needed to complete either LS or MC. The air samplers were connected to an AirChek XR5000 air pump (SKC) using an air flow of 2 (IOM) or 4 (Button) litres per minute depending upon the inhalable sampler used. Airflow was pre‐ and post‐calibrated using a rotameter (VFB‐65; Dwyer, Michigan City, IN, USA) calibrated to a primary airflow standard (Bios DryCal Defender 510; Mesa Labs, Butler, NJ, USA). After sampling, filters were stored in 50 ml polystyrene conical tube (Fisher Scientific) and stored at −20°C until further processing. Inhalable dust concentrations were determined gravimetrically.

### Genomic DNA extraction

The inhalable dust and settled dust were allowed to equilibrate to room temperature. Genomic DNA was extracted from samples having a minimum of 0.500 mg of dust on the filters (Table S2). The samples were added to the tubes of a commercial DNA extraction kit, PowerSoil DNA Isolation Kit (MoBio Laboratories, Carlsbad, CA, USA). Genomic DNA was extracted per manufacturer's instructions. Contaminants were removed using Agencourt Ampure XT Beads (Beckman Coulter, Pasadena, CA, USA). Genomic DNA was quantified using Quant‐iT PicoGreen dsDNA assay kit (Life Technologies, Carlsbad, CA, USA). All dust samples had genomic DNA quantity above the required 1 ng for Illumina 2500 library preparation (Table S2).

### Whole genome sequencing

Twenty‐one dual‐indexed libraries were prepared using Nextera XT DNA library prep kit (Illumina, San Diego, CA, USA) per manufacturers’ instructions. Libraries were combined into a single pool sequenced using a 125 bp paired‐end run on a HiSeq 2500 instrument (Illumina) using version 4 chemistry located at the University of Minnesota Genomics Center (Minneapolis, MN, USA). Lane yields were greater than 220 M reads/lane. All expected barcodes were detected in the demultiplexing report and were well balanced. Library inserts averaged 500 bp. Sequence data used in this analysis have been deposited to the NCBI Sequence Read Archive (Study Accession SRP075218) (Table S3).

### Sample genomic composition analysis

FastQ Screen (Babraham Bioinformatics; http://www.bioinformatics.babraham.ac.uk) was used to identify the sequence composition of the raw data using the bowtie2 aligner (Langmead and Salzberg, 2012) with a 500 000 sequence subset and default parameters. Reference genomes for human (GRCh37), chicken (galGal4), corn (AGPv3), soybean (GM01) and phage PhiX (1993‐04‐28) were downloaded from iGenomes (https://support.illumina.com/sequencing/sequencing_software/igenome.html). The rRNA reference database was downloaded from greengenes (http://greengenes.lbl.gov), and adapter sequences were collated from publicly available Illumina protocols for MiSeq and HiSeq experiments (Illumina). Although FastQ Screen identified non‐microbial, non‐adapter sequences present at low levels in the samples, these were not specifically filtered as the probability of aligning to the microbial clade‐specific reference databases used for downstream analysis was negligible (Segata *et al*., 2012).

### WGS read quality control

The FastQC tool (Babraham Bioinformatics; http://www.bioinformatics.babraham.ac.uk) was used to assess sequence quality for Illumina paired‐end dataset prior to downstream analysis. Data for each sample consisted of forward and reverse reads of length 126 nt and average GC content of 46.2%. Read quality was generally very high at all positions within the read. The introduction of sequencing errors towards the end of Illumina short reads can be a concern for accurate taxonomic classification (Camarinha‐Silva *et al*., 2014). The marker gene approach applied in this work relies on many dozens of markers to identify each species, making the introduction of random sequencing errors near the end of reads less of a concern as compared with methods that rely on one region to assign taxonomic classification. Thus, we decided not to truncate reads unless quality scores were below a threshold value (described below). FastQC identified adapter contamination from the Nextera XT transposase kit (Illumina). Forward and reverse reads were left unpaired, but were concatenated together into a single FASTQ file for quality control processing and downstream analysis. Trimmomatic (Bolger *et al*., 2014) was used to remove adapter contamination from the reads as well as further improve the mean quality scores across all reads prior to taxonomic profiling. The software settings were ‘ILLUMINACLIP:./adapters.fa:2:30:10’ to remove the Nextera adapter sequences and ‘LEADING:3 TRAILING:3 SLIDINGWINDOW:4:20’ to improve mean sequence quality by trimming leading and lagging bases with Q < 3 and any sequences with a four‐base sliding window mean below Q20.

### Taxonomic abundance profiling of WGS reads

Microbial relative abundance profiles were generated with MetaPhlAn2 (Segata *et al*., 2012) using default parameters and bowtie2 alignment on a single 10‐core node of an Intel(R) Xeon(R) E7‐4850 (2.0 GHz) server with 120GB of physical (RAM) memory. A single lane of forward and reverse reads (~13 million read pairs) was provided as input for each sample to MetaPhlan2. The MetaPhlAn2 reference database consists of clade‐specific marker genes (*i.e.,* genes that unambiguously characterize a taxonomic clade as they are always present in the sequenced isolates of that clade and never present in any other sequenced organism) from ~17 000 reference genomes (79% bacteria/archea, 20.4% viral and 0.6% eukaryotic). Profiles for all samples were merged with the script ‘merge_metaphlan_tables.py’ included with the MetaPhlAn2 distribution and heatmaps were generated with ‘metaphlan_hclust_heatmap.py’ script using default options and the ‘‐d braycurtis’, ‘‐minv 0.01’, ‘‐c bbcyr’ flags. Pie plots were generated with the script ‘metaphlan2krona.py’ and the KronaTools tool suite (Ondov *et al*., 2011).

### Principal component analysis

Principal component analysis of the sample abundance profiles was carried out by first merging MetaPhlan2 results into a single BIOM format table. A Qiime (Caporaso *et al*., 2010) utility script for calculating the Bray‐Curtis (Bray and Curtis, 1957) dissimilarity matrix on BIOM‐formatted taxonomic profiles was then used. The resulting dissimilarity matrix was imported into Python; calculation of PCA was performed with the scikit‐learn ‘decomposition’ library (Pedregosa *et al*., 2011). The first principal component contained 65% of the variation and the second principal component contained 9% of the variation in the data. Graphical analysis was performed in Python.

### Statistical biomarker analysis

A statistical analysis of differentially abundant features between classes of samples was accomplished with the LEfSe statistical analysis package. A table of species abundances for all samples was first prepared for analysis using the ‘format_input.py’ script with the ‘‐o 1000000’ option to normalize all values to one million as recommended by the package authors. Three classes were defined as LS (15 samples), MC (3 samples) and S (3 samples). Running the analysis was accomplished with the ‘run_lefse.py’ script using a default *P*‐value of 0.05 and the ‘‐y l’ option to specify the ‘all‐versus‐all’ comparison for the initial class‐level Kruskal–Wallis test prior to model building with LDA (Segata *et al*., 2011). The internal Wilcoxon test on subclasses was not performed because no subclasses were defined in the data. The cut‐off value of three or greater for the log LDA effect size is arbitrary, selected to limit the features identified by the analysis to a reasonable number of the most significant features for plotting.

## Supporting information


**Table S1** *Environmental and Chicken House Conditions at Time of Sampling*. During litter sampling and mortality collection, personal exposure to inhalable dust was collected using a personal inhalable dust sampler at a flow rate of 4 L/min. Environmental and house conditions were recorded at time of sampling.
**Table S2** *Genomic DNA quantity and total bases sequenced by Illumina 2500 in Poultry Dust Samples*. Dust concentrations of personal inhalable dust collected during litter sampling (LS) and mortality collection (MC) were determined through gravimetrically analysis of polyvinyl chloride filters. Genomic DNA was extracted from LS, MC, and settled (S) poultry dust using PowerSoil DNA Isolation Kit. Genomic DNA was quantified using the Quant‐iT PicoGreen dsDNA assay kit. Total reads and total gigabases (Gb) sequenced from the poultry dust samples were obtained through de novo synthesis of genomic DNA using 125 bp paired‐end Illumina 2500 instrument. *Only one lane out of five lanes were used for data analysis. Therefore, approximately 13 million reads were used for assembly.
**Table S3** *Biosample Accessions Numbers of Sequencing Data uploaded into the NCBI Biosample Database*. Sequence data used in this analysis have been deposited to the NCBI Sequence Read Archive (Study Accession SRP075218). The sequencing data was uploaded under the sample number, which corresponds to the following Sample ID in the manuscript.
