# Supplementary material for: High throughput genomic sequencing of bioaerosols in broiler chicken production facilities
Source: Microb Biotechnol. 2016 Jul 28;9(6):782–91. doi: 10.1111/1751-7915.12380 (PMC5072194; doi:10.1111/1751-7915.12380)
Supplement: Supplementary file 1 — Table S1 Environmental and Chicken House Conditions at Time of Sampling. During litter sampling and mortality collection, personal exposure to inhalable dust was collected using a personal inhalable dust sampler at a flow rate of 4 L/min. Environmental and house conditions were recorded at time of sampling. Table S2 Genomic DNA quantity and total bases sequenced by Illumina 2500 in Poultry Dust Samples. Dust concentrations of personal inhalable dust collected during litter sampling (LS) and mortality collection (MC) were determined through gravimetrically analysis of polyvinyl chloride filters. Genomic DNA was extracted from LS, MC, and settled (S) poultry dust using PowerSoil DNA Isolation Kit. Genomic DNA was quantified using the Quant‐iT PicoGreen dsDNA assay kit. Total reads and total gigabases (Gb) sequenced from the poultry dust samples were obtained through de novo synthesis of genomic DNA using 125 bp paired‐end Illumina 2500 instrument. *Only one lane out of five lanes were used for data analysis. Therefore, approximately 13 million reads were used for assembly. Table S3 Biosample Accessions Numbers of Sequencing Data uploaded into the NCBI Biosample Database. Sequence data used in this analysis have been deposited to the NCBI Sequence Read Archive (Study Accession SRP075218). The sequencing data was uploaded under the sample number, which corresponds to the following Sample ID in the manuscript. [file MBT2-9-782-s001.docx]

**Supporting Information**

| Sample ID | Litter Treatment | Outdoor Temperature (°C) | Outdoor Relative Humidity (%) | Indoor Temperature (°C) | Indoor Relative Humidity (%) | Age of Birds (days) |
| --- | --- | --- | --- | --- | --- | --- |
| LS1 | NaHSO_4_ | 23 | N/A | 27 | N/A | 28 |
| LS2 | None | 18 | 59 | 27 | 65 | 9 |
| LS3 | NaHSO_4_ | 20 | 54 | 28 | 72 | 12 |
| LS4 | None | 25 | 44 | 27 | 77 | 13 |
| LS5 | None | 22 | 54 | 23 | 73 | 21 |
| LS6 | NaHSO_4_ | 16 | 63 | 24 | 79 | 21 |
| LS7 | None | 17 | 57 | 25 | 98 | 14 |
| LS8 | NaHSO_4_ | 24 | 38 | 27 | 81 | 14 |
| LS9 | NaHSO_4_ | 27 | 26 | 26 | 48 | 19 |
| LS10 | NaHSO_4_ | 23 | 34 | 27 | 53 | 13 |
| LS11 | NaHSO_4_ | 25 | 32 | 24 | 69 | 13 |
| LS12 | NaHSO_4_ | 29 | 28 | 24 | 56 | 17 |
| LS13 | NaHSO_4_ | 44 | 21 | 25 | 77 | 8 |
| LS14 | NaHSO_4_ | 25 | 34 | 25 | 34 | 26 |
| LS15 | NaHSO_4_ | 27 | 73 | 28 | 76 | 15 |
| S1 | H_2_SO_4_+Al_2_(SO_4_)_3_-14H_2_O (l) | 11 | 53 | 16 | 64 | 60 |
| S2 | H_2_SO_4_+Al_2_(SO_4_)_3_-14H_2_O (l) | 11 | 53 | 16 | 64 | 60 |
| S3 | H_2_SO_4_+Al_2_(SO_4_)_3_-14H_2_O (l) | 11 | 53 | 16 | 64 | 60 |
| MC1 | NaHSO_4_ | 36 | 21 | 30 | 52 | 36 |
| MC2 | NaHSO_4_ | 36 | 21 | 30 | 52 | 43 |
| MC3 | NaHSO_4_ | 36 | 21 | 30 | 52 | 48 |

**Table 1** *Environmental and Chicken House Conditions at Time of Sampling.* During litter sampling and mortality collection, personal exposure to inhalable dust was collected using a personal inhalable dust sampler at a flow rate of 4 L/min. Environmental and house conditions were recorded at time of sampling.

LS: Litter sampling

S: Settled dust

MC: Mortality Collection

NaHSO_4_: Sodium Bisulfate Poultry Litter Treatment

H_2_SO_4_+Al_2_(SO_4_)_3_-14H_2_O (l): Acidified liquid aluminum sulfate

**Table 2** *Genomic DNA quantity and total bases sequenced by Illumina 2500 in Poultry Dust Samples.* Dust concentrations of personal inhalable dust collected during litter sampling (LS) and mortality collection (MC) were determined through gravimetrically analysis of polyvinyl chloride filters. Genomic DNA was extracted from LS, MC, and settled (S) poultry dust using PowerSoil DNA Isolation Kit. Genomic DNA was quantified using the Quant-iT PicoGreen dsDNA assay kit. Total reads and total gigabases (Gb) sequenced from the poultry dust samples were obtained through de novo synthesis of genomic DNA using 125 bp paired-end Illumina 2500 instrument. ^*^Only one lane out of five lanes were used for data analysis. Therefore, approximately 13 million reads were used for assembly.

| Sample ID | Total Dust (mg) | Total gDNA extracted (ng) | Total Reads^*^ | Total Gb^*^ |
| --- | --- | --- | --- | --- |
| LS1 | 0.530 | 4.0 | 66560816 | 16.6 |
| LS2 | 0.506 | 9.0 | 57809540 | 14.5 |
| LS3 | 0.858 | 22 | 59418318 | 14.9 |
| LS4 | 0.561 | 7.0 | 56202539 | 14.1 |
| LS5 | 0.688 | 11 | 75369678 | 18.8 |
| LS6 | 0.799 | 13 | 64309827 | 16.1 |
| LS7 | 0.732 | 18 | 53460114 | 13.4 |
| LS8 | 1.072 | 23 | 48688407 | 12.2 |
| LS9 | 0.610 | 12 | 69661497 | 17.4 |
| LS10 | 0.584 | 4.0 | 82255864 | 20.6 |
| LS11 | 0.770 | 13 | 56118411 | 14.0 |
| LS12 | 0.690 | 2.0 | 57249187 | 14.3 |
| LS13 | 0.532 | 5.0 | 68985082 | 17.2 |
| LS14 | 0.516 | 8.0 | 67003607 | 16.8 |
| LS15 | 0.594 | 8.0 | 73764044 | 18.4 |
| S1 | 2.184 | 85 | 63711141 | 15.9 |
| S2 | 1.343 | 44 | 82256962 | 20.6 |
| S3 | 2.183 | 110 | 72514558 | 18.1 |
| MC1 | 4.635 | 27 | 53830126 | 13.5 |
| MC2 | 2.621 | 79 | 45203716 | 11.3 |
| MC3 | 2.378 | 68 | 53178889 | 13.3 |

LS: Litter sampling

S: Settled dust

MC: Mortality Collection

**Table 3** *Biosample Accessions Numbers of Sequencing Data uploaded into the NCBI Biosample Database.* Sequence data used in this analysis have been deposited to the NCBI Sequence Read Archive (Study Accession SRP075218). The sequencing data was uploaded under the sample number, which corresponds to the following Sample ID in the manuscript.

| Sample ID | Sample Name | Accession Number | BioProject ID | Taxonomy ID |
| --- | --- | --- | --- | --- |
| LS1 | 16_TAAGGCGA-TATCCTCT_L004 | SAMN05001533 | 321423 | 256318 |
| LS2 | 27_CGTACTAG-TATCCTCT_L004 | SAMN05001534 | 321423 | 256318 |
| LS3 | 29_AGGCAGAA-TATCCTCT_L004 | SAMN05001535 | 321423 | 256318 |
| LS4 | 30_TCCTGAGC-TATCCTCT_L004 | SAMN05001536 | 321423 | 256318 |
| LS5 | 33_GGACTCCT-TATCCTCT_L004 | SAMN05001537 | 321423 | 256318 |
| LS6 | 34_TAGGCATG-TATCCTCT_L004 | SAMN05001538 | 321423 | 256318 |
| LS7 | 35_CTCTCTAC-TATCCTCT_L004 | SAMN05001539 | 321423 | 256318 |
| LS8 | 36_CAGAGAGG-TATCCTCT_L004 | SAMN05001540 | 321423 | 256318 |
| LS9 | 37_GCTACGCT-TATCCTCT_L004 | SAMN05001541 | 321423 | 256318 |
| LS10 | 44_CGAGGCTG-TATCCTCT_L004 | SAMN05001542 | 321423 | 256318 |
| LS11 | 45_AAGAGGCA-TATCCTCT_L004 | SAMN05001543 | 321423 | 256318 |
| LS12 | 46_GTAGAGGA-TATCCTCT_L004 | SAMN05001544 | 321423 | 256318 |
| LS13 | 52_TAAGGCGA-AGAGTAGA_L004 | SAMN05001545 | 321423 | 256318 |
| LS14 | 53_CGTACTAG-AGAGTAGA_L004 | SAMN05001546 | 321423 | 256318 |
| LS15 | 66_AGGCAGAA-AGAGTAGA_L004 | SAMN05001547 | 321423 | 256318 |
| MC1 | DB62_CTCTCTAC-AGAGTAGA_L004 | SAMN05001548 | 321423 | 256318 |
| MC2 | DB68_CAGAGAGG-AGAGTAGA_L004 | SAMN05001549 | 321423 | 256318 |
| MC3 | DB72_GCTACGCT-AGAGTAGA_L004 | SAMN05001550 | 321423 | 256318 |
| S1 | PD1_TCCTGAGC-AGAGTAGA_L004 | SAMN05001551 | 321423 | 256318 |
| S2 | PD2_GGACTCCT-AGAGTAGA_L004 | SAMN05001552 | 321423 | 256318 |
| S3 | PD3_TAGGCATG-AGAGTAGA_L004 | SAMN05001553 | 321423 | 256318 |

LS: Litter sampling

S: Settled dust

MC: Mortality Collection
